# Partnership as a modulator of the effects of hormone replacement therapy in menopause

**DOI:** 10.3389/fgwh.2026.1775422

**Published:** 2026-06-02

**Authors:** Ludek Fiala, Jiří Lenz

**Affiliations:** 1 Faculty of Medicine in Pilsen, Charles University, Pilsen, Czechia; 2 First Faculty of Medicine, Charles University, Prague, Czechia; 3 General University Hospital in Prague, Prague, Czechia; 4 University Hospital Pilsen, Pilsen, Czechia; 5 Department of Pathology, Znojmo Hospital, Znojmo, Czechia; 6 University of Veterinary and Pharmaceutical Sciences Brno, Brno, Czechia

**Keywords:** hormone replacement therapy (HRT), MANSA, menopause, MRS, quality of life

## Abstract

**Objective:**

To evaluate the influence of partnership status on the perception of menopausal symptoms and quality of life in women undergoing hormone replacement therapy (HRT).

**Methods:**

The study included 60 menopausal women aged 40–85 years with significant climacteric symptoms. Participants were divided into two groups according to relationship status: women living in a stable partnership (*n* = 30) and women without a partner for at least one year (*n* = 30). Menopausal symptoms and quality of life were assessed using the Menopause Rating Scale (MRS) and the Manchester Short Assessment of Quality of Life (MANSA). All participants received combined HRT. Assessments were performed at baseline and after three months of therapy. Changes in symptom severity (ΔMRS) and quality of life (ΔMANSA) were analyzed, and Pearson correlation coefficients with corresponding *p*-values were used to assess relationships between variables.

**Results:**

HRT was associated with improvement in menopausal symptoms and quality of life in both groups. However, women living in a partnership showed a greater reduction in MRS scores and a greater increase in MANSA scores compared to women without a partner. After three months of HRT, statistically significant correlations (*p* < 0.05) between hormonal levels (estradiol, FSH), menopausal symptoms, and quality of life were observed exclusively in partnered women.

**Conclusion:**

HRT improves menopausal symptoms and quality of life; however, partnership status appears to play a moderating role. A stable partnership may facilitate the integration of psychobiological processes of hormonal changes and contribute to improved subjective adaptation to menopause.

## Introduction

Menopause represents a significant period in a woman's life, characterized by declining estrogen levels and progressive ovarian failure. These hormonal changes lead to a range of physical and psychological symptoms that may substantially affect daily functioning. The most common complaints include vasomotor symptoms, sleep disturbances, fatigue, anxiety, depressive symptoms, and sexual dysfunction. These manifestations can negatively impact occupational, social, and intimate life (1).

An important component of menopausal symptomatology is the genitourinary syndrome of menopause (GSM), which includes vaginal dryness, thinning of the vaginal epithelium, changes in the vulvovaginal microbiome, and urinary symptoms, in some cases associated with incontinence. These conditions may further impair quality of life and negatively affect intimate relationships (2).

One of the effective treatment options for menopausal symptoms is hormone replacement therapy (HRT), including local estrogen therapy, which has been shown to alleviate vasomotor and urogenital symptoms. Nevertheless, many women avoid HRT due to concerns about thromboembolic events or oncological risks, or because of contraindications (3).

The use of HRT has been historically controversial, particularly following the results of the Women's Health Initiative (WHI), which raised concerns regarding cardiovascular and oncological risks. Although subsequent analyses have refined these interpretations and highlighted the importance of individualized risk assessment, many women remain reluctant to initiate HRT due to persistent safety concerns. This ongoing debate continues to influence treatment decisions in clinical practice.

In such cases, antidepressants (particularly selective serotonin reuptake inhibitors) or local vaginal treatments are sometimes used, although their efficacy is often limited (4, 5).

In addition to biological factors, psychosocial factors such as social support, partnership, and quality of intimate relationships play a significant role in the experience of menopause. A partnership may serve as a source of emotional support, security, and stability, thereby shaping the subjective perception and interpretation of menopausal symptoms. The aim of this study was to evaluate differences in the perception of menopausal symptoms and quality of life between women living in a partnership and women without a partner during three months of hormone replacement therapy.

## Materials and methods

### Study groups and time points

Participants were stratified according to partnership status and assessed at two time points. Group A consisted of women living in a stable partnership, while Group B included women living without a partner for at least one year. Each group was evaluated at baseline prior to initiation of hormone replacement therapy (T0) and after three months of continuous treatment (T1).

This design resulted in four analytical conditions: partnered women at baseline (Group A–T0), partnered women after treatment (Group A–T1), non-partnered women at baseline (Group B–T0), and non-partnered women after treatment (Group B–T1). Longitudinal changes within groups and differences between groups were analyzed accordingly.

### Study design and ethical approval

This study was designed as a prospective pilot observational study aimed at exploring psychosocial moderators of the effects of hormone replacement therapy in menopausal women. The study protocol was approved by the Ethics Committee of the Faculty of Medicine, Charles University in Prague. All participants provided written informed consent prior to inclusion.

Due to the pilot nature of the study and ethical considerations, a deliberately limited sample size was selected, consisting of 60 women equally divided into two groups according to partnership status.

**Figure 1:**
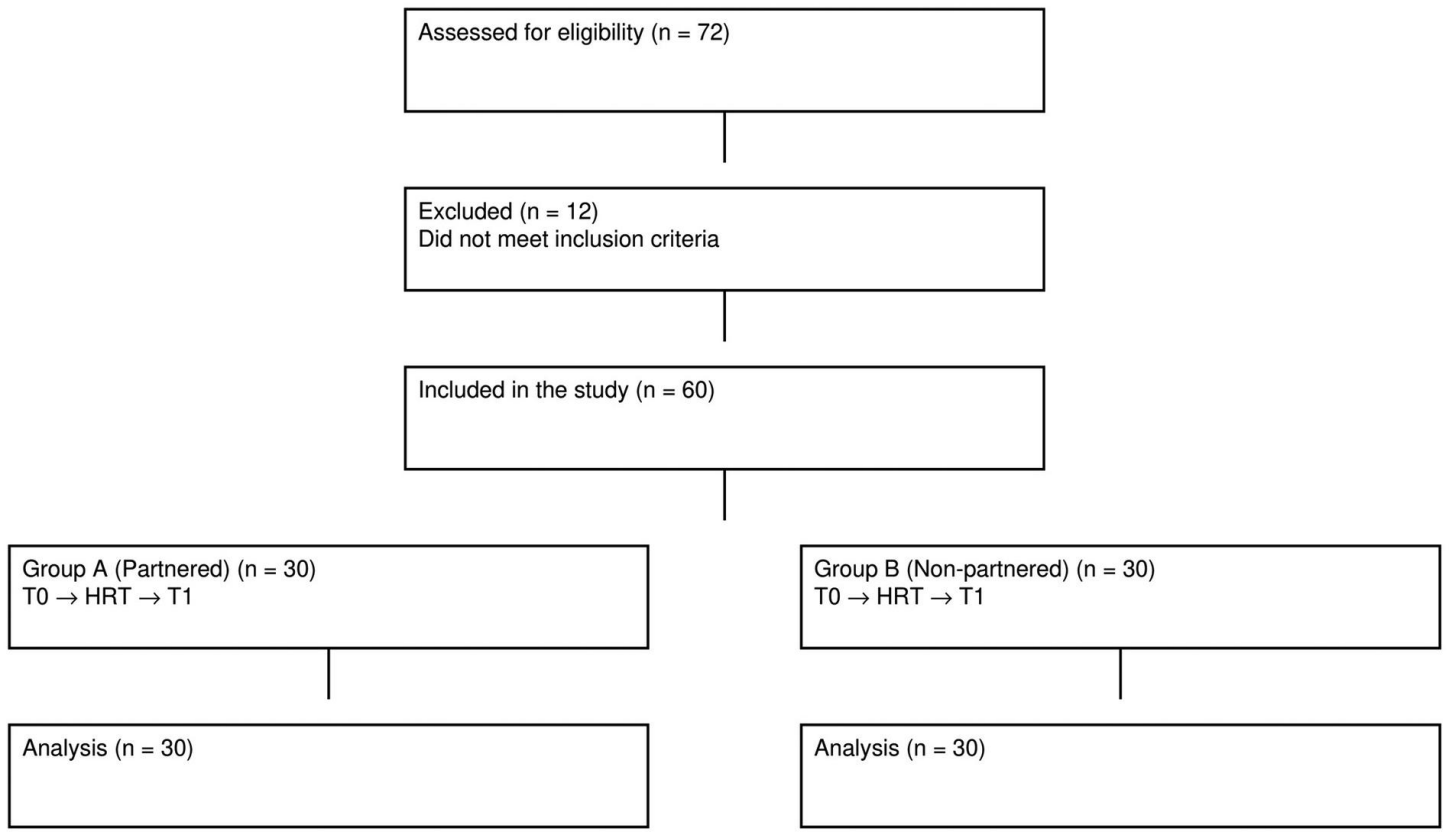
HRT effects in women with and without partners.

The flow of participants, including inclusion, exclusion, and group allocation, is illustrated in Figure 1.

### Assessed variables

Menopausal symptoms were assessed using the Menopause Rating Scale (MRS) (6), and quality of life was measured using the Manchester Short Assessment of Quality of Life (MANSA) (7). Biochemical parameters included serum estradiol and follicle-stimulating hormone (FSH) levels. Microbiological examination of the vaginal environment was performed at both baseline and follow-up visits.

### Outcomes

Primary outcomes were changes in symptom severity expressed as ΔMRS (MRS_T1 − MRS_T0) and changes in quality of life expressed as ΔMANSA (MANSA_T1 − MANSA_T0). Secondary outcomes included changes in hormonal parameters (Δestradiol, ΔFSH) and differences in treatment response between groups according to partnership status.

### Participants

Initially, 72 menopausal women with significant climacteric symptoms were screened. Inclusion criteria were age over 40 years and presence of menopausal symptoms. Exclusion criteria included a history of gynecological malignancy, including breast cancer, and current treatment with anxiolytics or antidepressants. Based on these criteria, 12 women were excluded.

In addition to inclusion and exclusion criteria, basic demographic data such as age were recorded. However, other potential confounding factors known to influence menopausal symptoms and quality of life—such as smoking status, psychosocial stress, and medical comorbidities—were not systematically collected. These variables may have influenced the observed outcomes and are addressed as a limitation of the study.

Menopausal status was confirmed by FSH levels > 40 IU/L. Estradiol levels typically range from 70 to 1,200 pmol/L in perimenopause and 50–70 pmol/L in postmenopause. The final analysis included 60 women divided into two groups:

- Group A (*n* = 30): women living in a partnership for at least one year (see Table 1)
- Group B (*n* = 30): women living without a partner for at least one year (see Table 2)

**Table 1:** Baseline characteristics (T0) by partnership status (mean ± SD).

Variable	Group A (Partnered) T0 (*n* = 30)	Group B (Non-partnered) T0 (*n* = 30)
Age (years)	64.33 ± 8.47	64.27 ± 8.61
MRS total score (0–44)	27.73 ± 7.65	28.77 ± 7.17
MANSA total score (16–112)	23.73 ± 4.02	21.77 ± 3.90
FSH (IU/L)	62.41 ± 18.07	62.71 ± 18.08
Estradiol, E2 (pmol/L)	157.00 ± 160.13	120.19 ± 28.61

Higher MRS scores indicate greater menopausal symptom severity. Higher MANSA total scores indicate better quality of life.

**Table 2:** Outcomes after 3 months of HRT (T1) by partnership status (mean ± SD).

Variable	Group A (Partnered) T1 (*n* = 30)	Group B (Non-partnered) T1 (*n* = 30)
Age (years)	64.33 ± 8.47	64.27 ± 8.61
MRS total score (0–44)	23.00 ± 8.94	26.77 ± 8.09
MANSA total score (16–112)	54.97 ± 17.24	21.50 ± 3.49
FSH (IU/L)	62.22 ± 17.68	62.42 ± 17.87
Estradiol, E2 (pmol/L)	171.69 ± 58.10	143.50 ± 29.96

Higher MRS scores indicate greater menopausal symptom severity. Higher MANSA total scores indicate better quality of life.

### Intervention

All participants were prescribed combined hormone replacement therapy in accordance with current clinical guidelines and received continuous treatment for three months. The specific formulation and dosage were individualized based on clinical indications and tolerability.

### Assessment instruments

The Menopause Rating Scale (MRS) is an internationally validated five-point Likert scale assessing the severity of menopausal symptoms. It consists of 11 items evaluating somatic, psychological, and urogenital symptoms, each scored from 0 (no symptoms) to 4 (very severe symptoms).

Interpretation: Score 0–44. 0–4 no or minimal symptoms; 5–8 mild symptoms; 9–16 moderate symptoms; ≥ 17 severe symptoms.

The Manchester Short Assessment of Quality of Life (MANSA) is a 16-item questionnaire assessing subjective quality of life, with items rated on a scale from 1 (worst possible state) to 7 (best possible state).

Interpretation: Score 0–112, values below 4.0 indicate low quality of life, values ≥ 6 indicate better quality of life.

### Biochemical analysis

All biochemical analyses were performed in an accredited clinical laboratory using standardized procedures. Detailed intra-assay and inter-assay coefficients were not available for this study.

Serum samples (2 mL) were obtained by standard venipuncture into vacuum tubes with gel separators. Samples were transported within 20 min at 4 °C to the Central Laboratory of the Institute of Medical Biochemistry and Laboratory Diagnostics, Faculty of Medicine in Prague. Estradiol and FSH levels were measured at baseline and after completion of the three-month HRT period (8).

### Statistical analysis

Statistical analyses were performed using STATISTICA software, version 12. Changes in MRS and MANSA scores (ΔMRS and ΔMANSA) were compared between groups using appropriate parametric tests (independent and paired t-tests, as applicable). Pearson's correlation coefficient was used to assess relationships between hormonal levels, menopausal symptoms, and quality of life.

Statistical significance was defined as *p* < 0.05.

## Results

Outcomes after three months of HRT are presented in Table 2. Both groups showed improvement in menopausal symptoms and quality of life; however, the magnitude of change differed between groups. Changes from baseline (ΔMRS and ΔMANSA) are summarized in Table 3 and indicate a greater treatment response in partnered women.

**Table 3:** Change from baseline to 3 months (Δ = T1 − T0), mean change.

Variable	Δ Group A (Partnered)	Δ Group B (Non-partnered)
ΔMRS (total score)	−4.73	−2.00
ΔMANSA (total score)	+31.23	−0.27
ΔFSH (IU/L)	−0.19	−0.29
ΔE2 (pmol/L)	+14.69	+23.31

Negative ΔMRS indicates symptom improvement. Positive ΔMANSA indicates improvement in quality of life.

After three months of HRT (Group A–T1), statistically significant correlations (*p* < 0.05) between selected clinical, psychological, and hormonal variables were observed exclusively in women living in a partnership.

Age showed a moderate positive correlation with menopausal symptom severity assessed by the MRS (*r* = 0.384). A moderate negative correlation was found between MRS scores and quality of life measured by MANSA (*r* = −0.415). A very strong positive correlation was observed between MRS scores and estradiol levels (*r* = 0.844). In addition, a moderate negative correlation was found between estradiol levels and quality of life (*r* = −0.515) (see Table 4).

**Table 4:** Correlation matrix after 3 months of HRT in partnered women (group A–T1).

Pearson correlation coefficient (r)				
	Age	MRS total	MANSA total	Estradiol (E2)
Age	1.000	0.384	—	—
MRS total	0.384	1.000	−0.415	0.844
MANSA total	—	−0.415	1.000	−0.515
Estradiol (E2)	—	0.844	−0.515	1.000

Values shown are Pearson's r for Group A at T1. Cells marked “—” were not reported. Correlations were not observed in the non-partnered group at T1, and no meaningful correlations were observed at baseline.

In women without a partner, no statistically significant correlations were observed (all *p* > 0.05) after three months of HRT between age, menopausal symptoms, quality of life, or hormonal parameters. Prior to initiation of HRT, no statistically significant correlations were found in either group (all *p* > 0.05).

## Discussion

Our findings indicate that relationships between hormonal changes, severity of menopausal symptoms, and subjective quality of life become structured after initiation of hormone replacement therapy only in women living in a partnership. While no significant correlations were present prior to treatment and variables remained uncorrelated in women without a partner even after three months of HRT, a consistent and statistically significant pattern of associations emerged in partnered women. This suggests that partnership status may influence not only subjective experience but also the integration of biological and psychological processes (9).

The very strong association between estradiol levels and MRS scores suggests that hormonal changes induced by HRT may not represent merely biological processes but acquire subjective significance reflected in the experience of menopausal symptoms. The concurrent negative correlation between estradiol levels and quality of life, together with the negative association between MRS and MANSA, supports the hypothesis that hormonal intervention influences quality of life indirectly through a complex psychobiological adaptation. It is important to emphasize that, in routine clinical practice, serum hormone levels are not typically used to guide HRT dosing, which is primarily adjusted according to symptom relief. Therefore, the observed associations between estradiol levels and clinical outcomes should not be interpreted as indicators of treatment adequacy but rather as exploratory biological correlates reflecting interindividual variability.

The fact that these relationships were observed exclusively in women living in a partnership points to a potential moderating role of psychosocial context. A partnership may provide a framework in which bodily and emotional changes are reflected upon, shared, and integrated into daily functioning, whereas in women without a partner, hormonal changes may remain psychologically isolated and less structured. These findings support a biopsychosocial conceptualization of menopause and suggest that the impact of HRT on subjective experience and quality of life is conditioned by relational context (10).

In addition, specific sexual dysfunctions such as hypoactive sexual desire disorder (HSDD), dyspareunia, or detailed manifestations of genitourinary syndrome of menopause were not evaluated as independent variables. Although some aspects were partially captured within the MRS scale, these factors may independently influence sexual function and quality of life and thus represent potential confounders of the observed associations.

### Limitations

Several limitations of this study should be acknowledged. First, due to its pilot design, the sample size was relatively small, limiting statistical power and generalizability. Second, the follow-up period was limited to three months, precluding assessment of long-term effects.

Third, although all participants received hormone replacement therapy, variability in formulations, routes of administration, and dosing was not systematically analyzed, which may have contributed to heterogeneity in treatment response.

Fourth, partnership status was assessed dichotomously without detailed evaluation of relationship quality, duration, or dynamics.

Finally, important potential confounding variables such as smoking status, psychosocial stress, occupational factors, and medical comorbidities were not systematically collected. These factors may influence menopausal symptoms and quality of life and should be addressed in future studies.

Given the exploratory and correlational nature of the study, these findings should be interpreted with caution and do not imply causal relationships. Rather, they provide a basis for future research exploring psychosocial moderators of hormonal treatment effects.

## Conclusion

This study suggests that the effects of hormone replacement therapy on menopausal symptoms and quality of life cannot be fully understood without considering the psychosocial context in which hormonal changes occur. Partnership appears to act as a moderating factor that may facilitate the integration of hormonal changes, symptom experience, and subjective well-being. These findings should be interpreted as exploratory and hypothesis-generating, highlighting the importance of incorporating relational and psychosocial factors into the clinical assessment and management of menopausal women undergoing HRT (11).

## Data Availability

The raw data supporting the conclusions of this article will be made available by the authors, without undue reservation.

## References

[1] Thornton K Chervenak J Neal-Perry G. Menopause and sexuality. Endocrinol Metab Clin North Am. (2015) 44(3):649–61. 10.1016/j.ecl.2015.05.009 26316248 PMC5994393

[2] Takahashi TA Johnson KM. Menopause. Med Clin North Am. (2015) 99(3):521–34. 10.1016/j.mcna.2015.01.006 25841598

[3] Gava G Orsili I Alvisi S Mancini I Seracchioli R Meriggiola MC. Cognition, mood and sleep in menopausal transition: the role of menopause hormone therapy. Medicina (Kaunas). (2019) 55(10):668. 10.3390/medicina55100668 31581598 PMC6843314

[4] Birkhäuser M. Climacteric depression and anxiety. Ther Umschau. (2021) 78(8):427–34. 10.1024/0040-5930/a001293 34555975

[5] Patel B S Dhillo W. Menopause review: emerging treatments for menopausal symptoms. Best Pract Res Clin Obstet Gynaecol. (2022) 81:134–44. 10.1016/j.bpobgyn.2021.10.010 34965909

[6] Honermann L Knabben L Weidlinger S Bitterlich N Stute P. An extended menopause rating scale II: a retrospective data analysis. Climacteric. (2020) 23(6):608–13. 10.1080/13697137.2020.1775808 32543231

[7] Priebe S Huxley P Knight S Evans S. Application and results of the Manchester short assessment of quality of life (MANSA). Int J Soc Psychiatry. (1999) 45(1):7–12. 10.1177/002076409904500102 10443245

[8] Fiala L Lenz J Bob P. Effect of psychosocial trauma and stress on sexual dysfunction in women with endometriosis. Medicine (Baltimore). (2021) 100(31):e26836. 10.1097/MD.0000000000026836 34397850 PMC8341311

[9] Ye L Knox B Hickey M. Management of menopause symptoms and quality of life during the menopause transition. Endocrinol Metab Clin North Am. (2022) 51(4):817–36. 10.1016/j.ecl.2022.04.006 36244695

[10] Kling JM Kelly M Rullo J Kapoor E Kuhle CL Vegunta S. Association between menopausal symptoms and relationship distress. Maturitas. (2019) 130:1–5. 10.1016/j.maturitas.2019.09.006 31706430

[11] Dennerstein L Alexander JL Kotz K. The menopause and sexual functioning: a review of the population-based studies. Annu Rev Sex Res. (2003) 14:64–82. 10.1080/10532528.2003.10559811 15287158

